# Use of conventional cardiac troponin assay for diagnosis of non-ST-elevation myocardial infarction: ‘The Ottawa Troponin Pathway’

**DOI:** 10.1371/journal.pone.0226892

**Published:** 2020-01-10

**Authors:** Venkatesh Thiruganasambandamoorthy, Ian G. Stiell, Hina Chaudry, Muhammad Mukarram, Ronald A. Booth, Cristian Toarta, Guy Hebert, Robert S. Beanlands, George A. Wells, Marie-Joe Nemnom, Monica Taljaard

**Affiliations:** 1 Department of Emergency Medicine, University of Ottawa, Ottawa, ON, Canada; 2 Ottawa Hospital Research Institute, The Ottawa Hospital, Ottawa, ON, Canada; 3 School of Epidemiology and Public Health, University of Ottawa, Ottawa, ON, Canada; 4 Department of Pathology and Laboratory Medicine, University of Ottawa, Ottawa, ON, Canada; 5 Division of Emergency Medicine, University of Toronto, Toronto, ON, Canada; 6 Division of Cardiology, University of Ottawa, Ottawa, ON, Canada; Azienda Ospedaliero Universitaria Careggi, ITALY

## Abstract

**Background:**

Serial conventional cardiac troponin (cTn) measurements 6–9 hours apart are recommended for non-ST-elevation MI (NSTEMI) diagnosis. We sought to develop a pathway with 3-hour changes for major adverse cardiac event (MACE) identification and assess the added value of the HEART [History, Electrocardiogram (ECG), Age, Risk factors, Troponin] score to the pathway.

**Methods:**

We prospectively enrolled adults with NSTEMI symptoms at two-large emergency departments (EDs) over 32-months. Patients with STEMI, unstable angina and one cTn were excluded. We collected baseline characteristics, Siemens Vista conventional cTnI at 0, 3 or 6-hours after ED presentation; HEART score predictors; disposition and ED length of stay (LOS). Adjudicated primary outcome was 15-day MACE (acute MI, revascularization, or death due to cardiac ischemia/unknown cause). We analyzed multiples of 99th percentile cut-off cTnI values (45, 100 and 250ng/L).

**Results:**

1,683 patients (mean age 64.7 years; 55.3% female; median LOS 7-hours; 88 patients with 15-day MACE) were included. 1,346 (80.0%) patients with both cTnI≤45 ng/L; and 155 (9.2%) of the 213 patients with one value≥100ng/L but both<250ng/L or ≤20% change did not suffer MACE. Among 124 patients (7.4%) with one of the two values>45ng/L but<100ng/L based on 3 or 6-hour cTnI, one patient with absolute change<10ng/L and 6 of the 19 patients with≥20ng/L were diagnosed with NSTEMI (patients with Δ10-19ng/L between first and second cTnI had third one at 6-hours). Based on the results, we developed the Ottawa Troponin Pathway (OTP) with a 98.9% sensitivity (95% CI 93.8–100%) and 94.6% specificity (95% CI 93.3–95.6%). Addition of the HEART score improved the sensitivity to 100% (95% CI 95.9–100%) and decreased the specificity to 26.5% (95% CI 24.3–28.7%).

**Conclusion:**

The OTP with conventional cTnI 3-hours apart, should lead to better NSTEMI identification particularly those with values >99th percentile, standardize management and reduce the ED LOS.

## Introduction

Chest pain is the second most common emergency department (ED) presenting complaint.[1] Chest pain constitutes approximately 5% of all ED visits translating to approximately 10 million ED visits in the US annually, and acute myocardial infarction (AMI) hospitalization is costly.[2, 3] One of the main objectives of ED evaluation of patients with chest pain or other anginal equivalent symptoms (e.g. shortness of breath, chest discomfort, indigestion) is to the rule-out acute coronary syndrome (ACS). ACS caused by acute myocardial ischemia, includes unstable angina, ST-elevation and non-ST elevation myocardial infarction (STEMI, NSTEMI), and is associated with substantial morbidity and mortality. Among those diagnosed with ACS, 70% have NSTEMI which are diagnosed using cardiac troponin (cTn) assays.[4] Approximately 85% of all patients presenting to the ED with chest pain will be discharged home.[5] Hence, accurate yet quick disposition decision for this common condition is a critical issue that affects ED crowding. High-sensitivity troponin (hsTn) assays are commonly used in Europe and some parts of North America. However, a substantial proportion of health systems worldwide are still using the conventional cTn assays. Additionally, some studies have reported loss of specificity with hsTn assays and lack of improvement in patient centered outcomes when compared to conventional cTn assays.[6, 7] Guidelines from professional societies are very conservative mandating serial conventional cTn testing, with second troponin measurement recommended at 6–9 hours after the first one.[8, 9] Secondary analyses of previous studies reported that a negative conventional cTn assay at 0 and 3 hours of ED presentation, defined as a value below the 99^th^ percentile cut-off, among low-risk chest pain patients could reliably rule out AMI.[10, 11] This has not been confirmed in prospective studies and no previous studies have explored the role of either absolute or relative changes with serial conventional cTn assay for diagnosis of NSTEMI among patients with levels above the 99^th^ percentile cut-off.

The overall objective of this study was to identify strategies to improve the safety and efficiency of ED management of patients with suspected NSTEMI with better use of the conventional cTn assay. The primary objective of the study was to develop a pathway based on absolute and relative changes between two cTn measurements performed 3-hours apart for accurate identification of patients at risk for 15-day major adverse cardiac event (MACE). The secondary objective was to evaluate the added value of the HEART (History, ECG, Age, Risk factors, Troponin) score to the pathway.

## Materials and methods

### Study setting and population

Our study was a prospective cohort study conducted at two-large academic Canadian EDs. We enrolled adults (age ≥ 16 years) with chest pain or non-chest pain symptoms within the past 24-hours among whom NSTEMI was a concern. We excluded patients with a STEMI diagnosis based on their presenting electrocardiogram (ECG) and those hospitalized with a diagnosis of unstable angina. We excluded patients who were hospitalized with a diagnosis of unstable angina as troponin values have no role in the disposition decision making for these patients. For the purposes of developing the pathway, we excluded patients who had only one troponin measurement performed during the ED visit. The study was conducted between March 2014 and October 2017.

### Study protocol

The usual management of patients with suspected NSTEMI at the study hospitals includes history, physical exam, ECG and cTn measurements. ED physicians at times performed only a single Tn measurement for patients with prolonged symptoms or if the patients presented late after the most recent episode of symptoms. If serial cTn measurements were performed, they were usually done 6–8 hours after the first cTn. As per the study protocol, we requested physicians to obtain an additional troponin level 3-hours after the first one. The timing and further troponin measurements beyond 3-hours of the first troponin was left to the discretion of the treating physician. The Ottawa Health Science Network Research Ethics Board at the study hospital approved the protocol with the requirement of only verbal patient consent.

### Data collection

We collected demographics, presenting symptoms (chest pain, shortness of breath, angina equivalent, non-cardiac symptoms, other cardiac symptoms–palpitations, syncope) and their characteristics, medical history, medications, results of blood tests including troponin, diagnosis at the end of the ED visit, and the final diagnosis based on outpatient investigations or consultations. Symptom characteristics including the time of the most recent episode, the number of episodes and the duration of symptoms was also collected. For troponin values, we collected time of the blood draw. Health resource utilization data such as time of ED arrival, referral to a consulting service, hospitalization, and time of ED discharge was also collected.

### Study outcomes

The primary outcome for this study was the occurrence of a MACE within 15-days of ED presentation and included AMI, revascularization, or death due to cardiac ischemia or an unknown cause. The outcome AMI included both STEMI that occurred after the index ED visit and NSTEMI both during or after the index ED visit. Patients presenting with STEMI during the index ED visit were excluded. Secondary outcomes included 30-day MACE, death not related to cardiac ischemia, and non-ACS outcomes related to the presenting symptom (e.g. serious valvular disease, pulmonary embolism).

We used a step-wise approach to ascertain the occurrence of study outcomes. First, we undertook a structured review of all available medical records at the study hospitals related to the index and subsequent ED visits, hospitalizations and/or death, and the results of all investigations including those performed in the outpatient setting. One of the two study sites is the only regional centre for the following advanced coronary artery disease investigations and interventions: coronary angiogram, angioplasty, stent placements and coronary by-pass grafting. As a second step, a scripted telephone follow-up was performed after 30 days. If the above two steps were unsuccessful, we searched obituaries for matching identification details. If no definite information was available with the above approaches, then the patient was classified as ‘lost to follow-up’.

NSTEMIs diagnosed during the ED or in-hospital stay needed to be confirmed by the consulting team. An adjudication committee comprised of two study physicians reviewed all study data including ED records, results of investigations, consultations performed and adjudicated the study outcomes independently. Disagreements were resolved by a third physician. We took into consideration the third universal definition of myocardial infarction when adjudicating the NSTEMI outcome among study patients.[12]

### Troponin assay at the study sites

Both study hospitals use the Siemens Vista cardiac troponin I (cTnI) assay which is classified as a conventional assay with respect to its sensitivity.[13] The lower limit of quantitation (LoQ) for this assay is 15 ng/L, the manufacturer recommended 99^th^ percentile for healthy population or the 99^th^ percentile of the upper reference limit (URL) is 45 ng/L. The co-efficient of variation (CV) at 50 ng/L is less than 10% and approximately 12% at 30 ng/L and is considered guideline acceptable.[13]

### Statistical analysis

We used mean, range and standard deviation, or median and inter-quartile range (IQR) as appropriate for continuous variables; and frequency with proportion for categorical variables. ED length of stay (LOS) was calculated as the time from ED arrival to ED discharge. For patients who had the second cTnI measurement done >3-hours after the first one and if below the 99^th^ percentile cut-off (<45ng/L), we assumed the value at the 3-hour mark was also <45ng/L. If the second cTnI measurement was performed between 2 hours 45 minutes and 3 hours 15 minutes, and the third cTnI between 5 hours 45 minutes and 6 hours 15 minutes, then the timings were considered appropriate for 3-hour and 6-hour cTnI measurements. Among patients with elevations in cTnI, our analytical plan included stratification based on multiples of 99^th^ percentile URL thresholds, and assessment of change using absolute values or relative variations. We chose stratification values of >45 ng/L (URL), >100ng/L and >250ng/L (approximately 2 and 5 times the URL), values of 10, 20 and 30 ng/L for absolute changes, and multiples of 10% for relative changes with 20% as the main relative change based on in-house assay precision (within run and between run), review of guidelines, review of troponin reference change value (RCV) reported in the literature and international recommendations.[9, 12, 14] We developed the Ottawa Troponin Pathway to achieve highest sensitivity based on two troponin values three hours apart. For the secondary objective, we retrospectively collected the HEART score predictors based on information documented in the patient chart to assess the performance of the pathway with the addition of the risk score.[15] We report diagnostic performance characteristics: sensitivity, specificity, positive and negative likelihood ratios for the overall pathway, its components, and report the any improvement in sensitivity and specificity with the addition of HEART score to the pathway. Where appropriate, we report 95% confidence intervals for the point estimates using either the large sample approximation or the exact binomial distribution when the expected number of events or non-events was less than 5. We used SAS (version 9.4) for data analysis.

## Results

**Fig 1:**
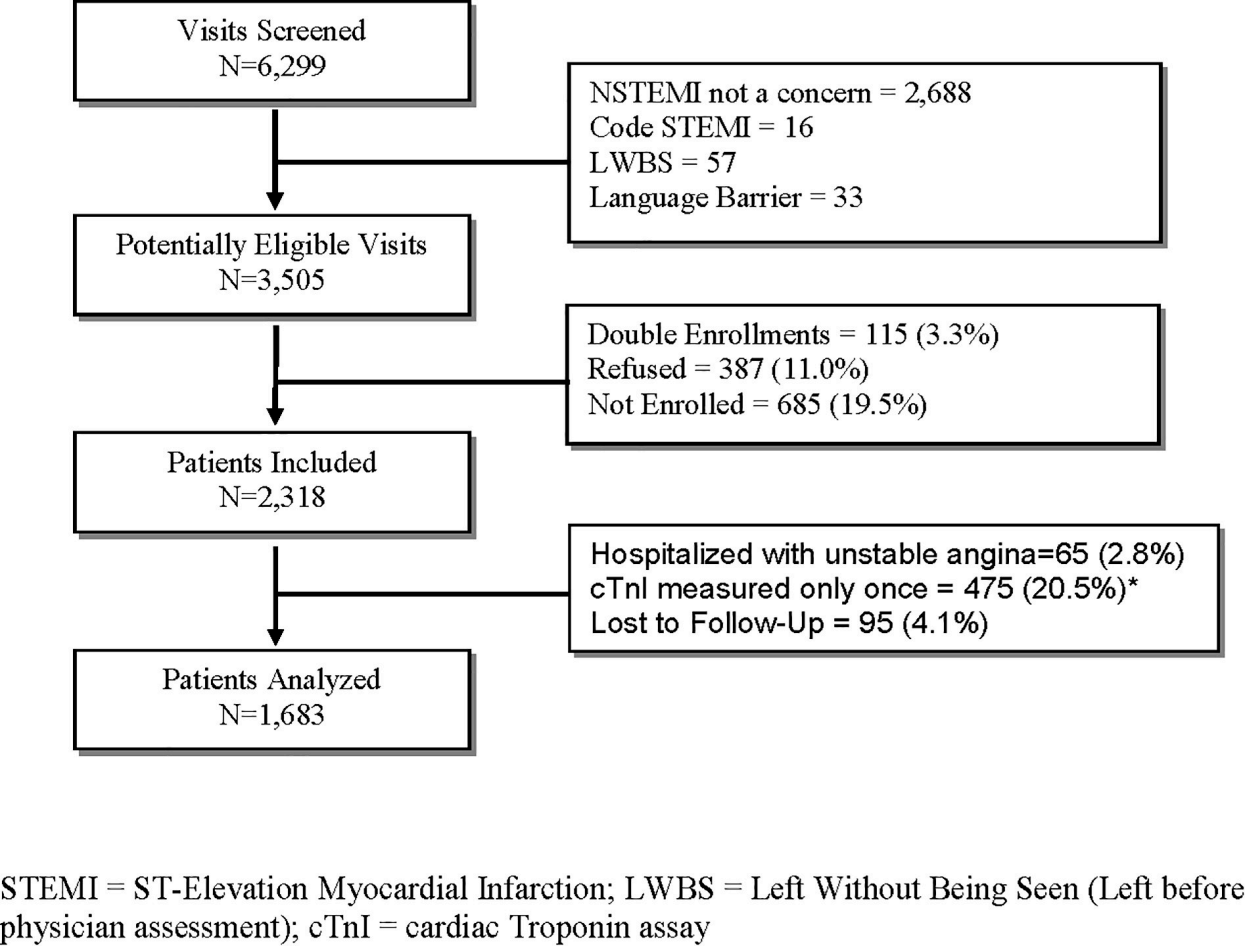
Patient flow. The median time to cTnI measurement from the time of onset of recent symptoms was 10.8 hours (IQR 4.4, 24.5 hours). Only one patient was diagnosed with NSTEMI based on high first TnI value and was hospitalized. The reminder did not suffer 30-day MACE. The median emergency department length of stay for these patients was 4 hours (IQR 3, 5 hours).

During the study period, 6,299 patients were screened, of whom 2,318 patients were enrolled in the study (Fig 1). A total of 65 patients (2.8%) in our study were hospitalized with unstable angina, 475 patients (20.5%) had only one troponin measurement during the ED visit, and the 30-day follow-up was incomplete for 95 patients (4.1%), leaving 1,683 patients for analysis. The characteristics of the study patients and their ED management are detailed in Table 1.

**Table 1:** Patient characteristics.

Demographics	N = 1,683
Age (in years), mean (SD)	64.7 (15.1)
Female, n (%)	930 (55.3)
Arrival by ambulance, n (%)	640 (38.0)
**Medical history, n (%)**	
Hypertension	870 (51.7)
Diabetes	332 (19.7)
Hyperlipidemia	621 (36.9)
Known coronary artery disease	579 (34.4)
Known congestive heart disease	147 (8.7)
Atrial Fibrillation/Flutter	235 (13.9)
Peripheral arterial disease	89 (5.3)
Known COPD	103 (6.1)
Pulmonary Embolism	25 (1.5)
Renal Failure on dialysis	22 (1.3)
**ED Management**	
Referred to consulting service, n (%)	343 (20.4)
Hospitalized, n (%)	199 (11.8)
ED Length of stay (in hours), median (IQR)	7 (6–9)
**15-Day Major Adverse Cardiac Events (MACE), n (%)**	
All MACE	88 (5.2)
Death due to cardiac or unknown cause	4* (0.1)
NSTEMI	86 (5.1)

COPD = Chronic Obstructive Pulmonary Disease; ED = Emergency Department; IQR = Interquartile Range; NSTEMI = Non-ST Elevation Myocardial Infarction; SD = Standard Deviation

*2 patients suffered NSTEMI (are listed under in the row NSTEMI) then died on day 2 and are not counted towards the total

Of the 1,683 patients included in the final analysis, 88 patients (5.2%; 95% CI 4.2%-6.4%) suffered 15-day MACE and included 86 patients with NSTEMI and 2 deaths due to cardiac ischemia or an unknown cause (Table 1; Table 2). None of the patients had revascularization as their primary outcome in the absence of NSTEMI diagnosis.

**Table 2:** 15-Day Major Adverse Cardiac Events (MACE)–N = 1,683.

Outcome, N (%)	Total	During the index ED visit		After the index ED visit
		In ED	As Inpatient	
Total	88 (5.2)	81 (4.8)	5 (0.3)	2 (0.1)
Death due to cardiac or unknown cause	4* (0.1)	0 (0.0)	3* (0.1)	1 (0.1)
NSTEMI	86 (5.1)	81 (4.8)	4 (0.2)	1 (0.1)

ED = Emergency Department; NSTEMI = Non-ST Elevation Myocardial Infarction

*2 patients suffered NSTEMI (are listed under in the row NSTEMI) then died on day 2 and are not counted towards the total

Of the 88 patients with 15-day MACE, while all were referred to a consulting service, 11 patients were assigned a different diagnosis than NSTEMI: 3 patients with atrial fibrillation rate related troponin rise; 6 were diagnosed as undifferentiated chest pain; one patient with each of three diagnoses–sepsis, cardiogenic shock, and non-ACS troponin rise. The patients who suffered 15-day MACE were slightly older, more likely to be women and with cardiovascular comorbidities (Table 3).

**Table 3:** Demographics for patients with Major Adverse Cardiac Events (MACE) at 15 days.

Demographics for patients with MACE at 15 days	N = 88
Age (in years), mean (SD)	68.5 (13.8)
Female, n (%)	60 (68.2)
Arrival by ambulance, n (%)	34 (38.6)
**Medical history, n (%)**	
Hypertension	59 (67.1)
Hyperlipidemia	55 (62.5)
Known coronary artery disease	46 (52.3)
Diabetes	27 (30.7)
Known congestive heart disease	11 (12.5)
Atrial Fibrillation/Flutter	10 (11.4)
Peripheral arterial disease	9 (10.2)
Known COPD	4 (4.6)
Renal Failure on dialysis	2 (2.3)
Pulmonary Embolism	1 (1.1)
**In ED**	
Referred to consulting service, n (%)	88 (100)
Hospitalized, n (%)	82 (93.2)
ED Length of stay (in hours), median (IQR)	5 (3–7)
First troponin value, median (IQR)	232 (46–1281.5)

ED = Emergency Department; COPD = Chronic Obstructive Pulmonary Disease; IQR = Interquartile Range; SD = Standard Deviation

### Development of the ottawa troponin pathway

**Fig 2:**
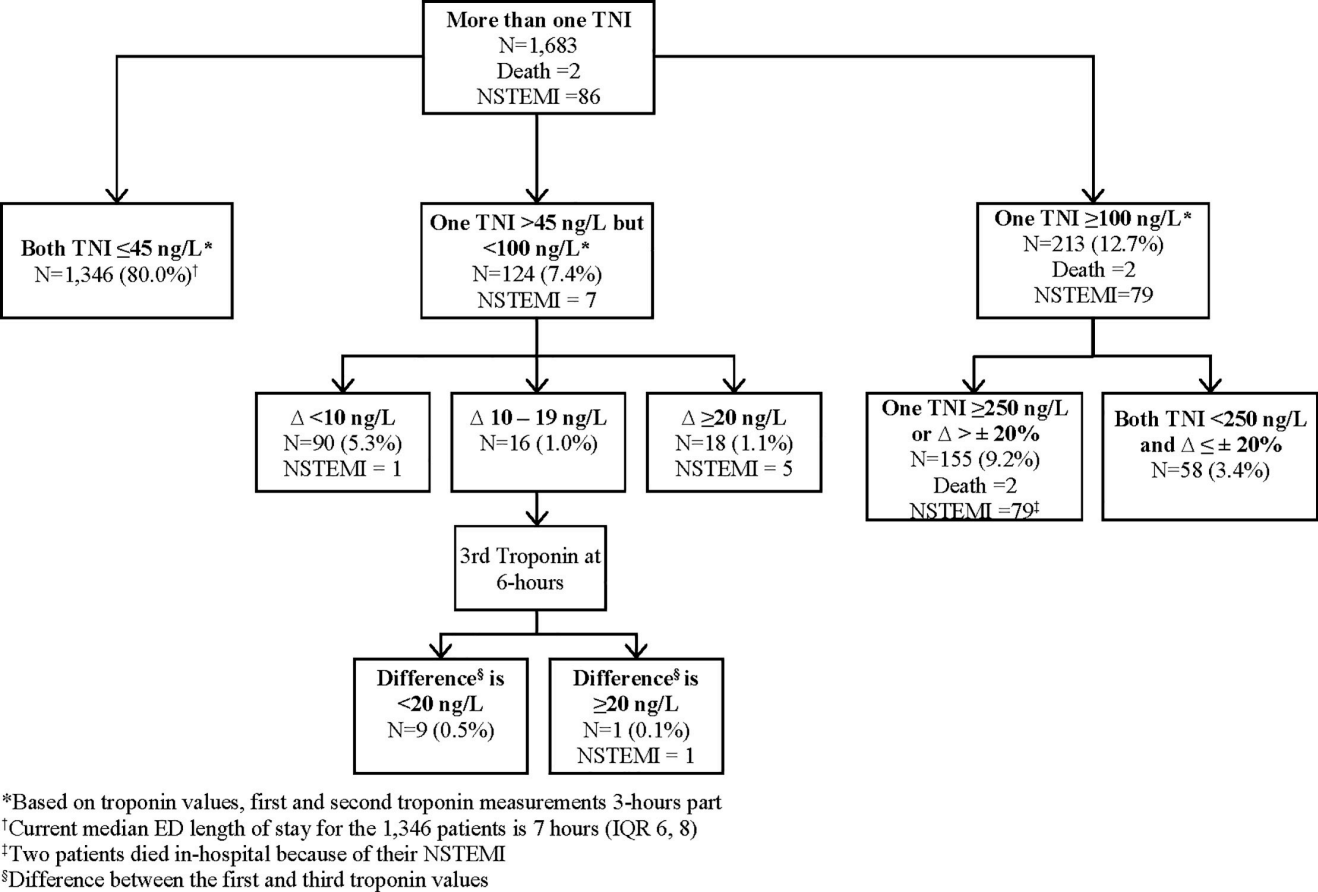
Classification of Patients based on Two or Three Serial Troponin Values and their 15-day Outcomes. *Based on troponin values, first and second troponin measurements 3-hours part †Current median ED length of stay for the 1,346 patients is 7 hours (IQR 6, 8) ‡Two patients died in-hospital due to NSTEMI §Difference between the first and third troponin values.

Overall, 1,683 patients in the study cohort had two or more cTnI measurements done with the time interval between the first and the second one around 3-hours apart. The flow of patients with two or more cTnI measurements stratified by threshold values of ≤45ng/L, >45 but <100ng/L, and ≥100ng/L; their absolute or relative changes; and their outcomes are detailed in Fig 2. The characteristics of patients designated to these groups, their ED management, outpatient investigations performed, final ED diagnosis and 15-day MACE outcomes are detailed in Table 4. Overall 5.6% of patients and 38.0% of patients in the >45 but <100ng/L and ≥100ng/L suffered 15-day MACE respectively. Review of the medical history of patients in the three groups did not elucidate clear reasons for cTnI elevations among those without MACE.

**Table 4:** Characteristics, management and outcomes for the three Ottawa Troponin Pathway groups.

Demographics	Both TNI ≤45 ng/L* N = 1,346	One TNI >45 ng/L but <100 ng/L* N = 124	One TNI ≥100 ng/L* N = 213
Age (in years), mean (SD)	63 (14.8)	72.3 (14.4)	70.6 (14.3)
Female, n (%)	736 (54.7)	71 (57.3)	123 (57.7)
Arrival by ambulance, n (%)	482 (35.8)	60 (48.4)	98 (46)
**Medical history, n (%)**			
Hypertension	653 (48.5)	77 (62.1)	140 (65.7)
Hyperlipidemia	238 (17.7)	41 (33.1)	53 (24.9)
Known coronary artery disease	458 (34)	54 (43.5)	109 (51.2)
Diabetes	418 (31.1)	58 (46.8)	103 (48.4)
Known congestive heart disease	77 (5.7)	30 (24.2)	40 (18.8)
Atrial Fibrillation/Flutter	158 (11.7)	32 (25.8)	45 (21.1)
Peripheral arterial disease	53 (3.9)	13 (10.5)	23 (10.8)
Known COPD	71 (5.3)	13 (10.5)	19 (8.9)
Renal Failure on dialysis	18 (1.3)	3 (2.4)	4 (1.9)
Pulmonary Embolism	8 (0.6)	5 (4)	9 (4.2)
**In ED**			
Referred to consulting service, n (%)	144 (10.7)	47 (37.9)	152 (71.4)
Hospitalized, n (%)	45 (3.3)	26 (21)	128 (60.1)
ED Length of stay (in hours), median (IQR)	7 (6–8)	8 (6–9)	6 (4–8)
**Final ED diagnosis**			
Acute myocardial infarction	5 (0.4)	10 (8.1)	78 (36.6)
Definite Unstable Angina	44 (3.3)	6 (4.8)	2 (0.9)
Probable Unstable Angina	21 (1.6)	2 (1.6)	5 (2.3)
Undifferentiated chest pain	807 (60.0)	43 (34.7)	37 (17.4)
Non-cardiac chest pain	357 (26.5)	33 (26.6)	35 (16.4)
Others†	108 (8.0)	28 (22.6)	55 (25.8)
**Outpatient investigations performed**	411 (30.5)	53 (42.7)	132 (62.0)
Stress testing	279 (20.7)	23 (18.5)	22 (10.3)
Cardiac Computed Tomography angiogram	29 (2.2)	1 (0.8)	0 (0)
Coronary angiography	51 (3.8)	18 (14.5)	89 (41.8)
**15-Day Major Adverse Cardiac Events (MACE), n (%)**			
All MACE	0 (0)	7 (5.6)	81 (38.0)
Death due to cardiac or unknown cause	0 (0)	0 (0)	2 (0.9)
NSTEMI	0 (0)	7 (5.6)	79 (37.1)

*Based on at least two TNI values measured during the index ED visit

†Others include a variety of cardiac and non-cardiac diagnosis such as congestive heart failure, arrhythmia, valvular heart disease, cardiomyopathy, pulmonary embolism, myocarditis, myopericarditis, pericarditis, pleural effusion, stable angina, implantable cardioverter defibrillator shocks, and pneumonia.

COPD = Chronic Obstructive Pulmonary Disease; ED = Emergency Department; IQR = Interquartile Range; MACE = Major Adverse Cardiac Events; NSTEMI = Non-ST Elevation Myocardial Infarction; TNI = Troponin; SD = Standard Deviation

A subgroup of 1,346 patients (80.0%) had two cTnI measurements performed around 3-hours apart and both values at or below the 99^th^ percentile URL (≤45ng/L). None of the 1,346 patients suffered MACE within 15-days. One hundred and ninety-six patients (14.6%) in this subgroup had the second cTnI measured before the 3-hour mark and none of them suffered MACE outcomes. One patient suffered 30-day secondary MACE outcome and was diagnosed with NSTEMI during a return ED visit on the 30^th^ day. A total of 699 patients (51.9%) in this subgroup did have a third cTnI measurement performed around 6 hours. The median ED LOS for this subgroup of patients was 7 hours (IQR 6, 8 hours).

There were 124 patients (7.4%) who had one of the two cTnI values >45ng/L but both values were <100ng/L. Of the 124 patients, 90 patients (72.6% of patients in this sub-group), had <10ng/L absolute change between the first two values and one patient suffered NSTEMI before 15-days. Eight patients who had <10ng/L change had their second cTnI measured before 3-hours and none suffered MACE. Eighteen patients (14.5% in this subgroup) had ≥20ng/L absolute change between the first and second cTnI values, of whom 5 patients suffered NSTEMI. Of the remaining 16 patients, a third troponin measurement was ordered for 10 patients. The absolute difference between the third and first cTnI values were still <20ng/L for 9 of these patients and none of them suffered a 30-day MACE outcome. The one patient who had an absolute difference between the third and the first cTnI ≥20ng/L was diagnosed with NSTEMI. Six patients did not have a third cTnI measurement performed and none suffered MACE within 30-days.

There were 213 (12.7%) patients who had one of the two cTnI values ≥100ng/L. Among this subgroup, 155 patients (72.8% in this subgroup) had one of the two levels ≥250ng/L or >20% rise or fall between the two cTnI, of whom 2 died due to unknown cause and 79 suffered NSTEMI. Of the 79 patients who suffered NSTEMI, 2 patients died in-hospital due to their NSTEMI. Twenty-seven patients (12.7% of this subgroup) had their second troponin performed before 3 hours, of whom 1 died and 11 suffered NSTEMI and were positive for the criteria. Of 58 patients who were negative for the criteria (any level ≥250ng/L or >20% change), none suffered MACE within 30-days. Among those who had cTnI performed early and were negative for the criteria, none suffered MACE within 30-days.

**Fig 3:**
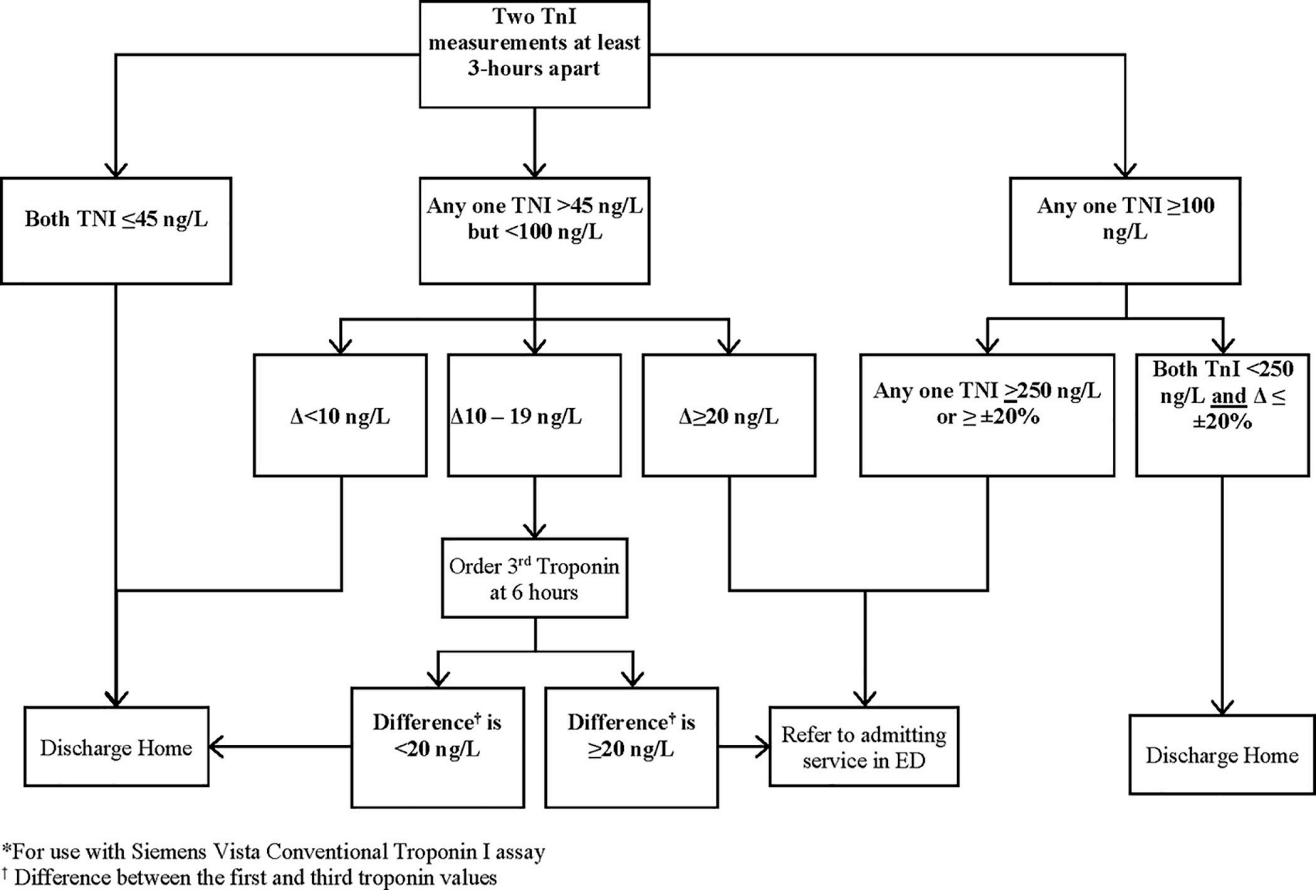
The Ottawa Troponin Protocol*. *For use with Siemens Vista Conventional Troponin I assay † Difference between the first and third troponin values.

Based on the above analysis, we developed the Ottawa Troponin Pathway (OTP; Fig 3) for troponin testing using the Siemens Vista conventional cTnI assay for patients with suspected NSTEMI. The diagnostic characteristics, sensitivity, specificity, positive and negative likelihood ratios with 95% CIs for the entire pathway and its components to identify 15-day MACE are detailed in Table 5. At the study hospitals, 343 patients (20.4%) were referred to the consulting service for ACS, and 2 patients suffered 15-day MACE outcomes after the index ED visit (Table 2) representing a sensitivity of 97.7% (95% CI 91.3%, 99.6%) and a specificity of 83.8% (95% CI 83.5%, 83.9%). The current ED LOS is 7 hours (IQR 6, 9 hours). The addition of the HEART score (cut off >3) improved the sensitivity of the pathway to 100% for 15-day MACE. However, the specificity dropped from 94.6% to 26.5% (Table 5).

**Table 5:** Diagnostic performance of the Ottawa Troponin Pathway and its components.

Troponin thresholds*	Total number of patients	No. of Patients fulfilling criteria	15-day MACE	Sensitivity % (95% CI)	Specificity % (95% CI)	Positive LR (95% CI)	Negative LR (95% CI)
Both ≤45ng/L*^§^	1,346	1,346	0	--	100 (99.7–100)	--	1.0
If any value >45 but <100ng/L* Δ ≥20ng/L^†^	124	19	7	85.7 (42.1–99.6)	88.9 (81.8–94.0)	7.7 (3.1–12.3)	0.2 (0.0–0.5)
If any value ≥100ng/L* Any one TNI ≥250 ng/L or Δ > ± 20%	213	155	81	100 (95.6–100)	43.9 (35.3–52.8)	1.8 (1.5–2.1)	0.0
Overall pathway performance	1,683	174	88	98.9 (93.8–100)	94.6 (93.3–95.6)	18.1 (14.4–21.9)	0.01 (0.0–0.04)
Pathway + HEART score >3	1,683	1261	88	100% (95.9–100)	26.5 (24.3–28.7)	1.3 (1.3–1.4)	0.0

CI = Confidence Interval; ED = Emergency Department; HEART = History, ECG, Age, Risk factors for atherosclerosis, and Troponin; MACE = Major Adverse Cardiac Event (death due to cardiac or an unknown cause); LR = Likelihood Ratio

*Based on two serial troponin measurements around 3-hours apart

^†^If the Δ between the first and second is between 10 and 19ng/L, then measure a third level at 6-hours; and the change is calculated between the first and third troponin values

^§^ Diagnostic tests and their 95% CIs could not all be calculated due to the absence of outcomes

### Secondary outcomes

In addition to the 88 patients who suffered 15-day MACE outcomes, one patient suffered 30-day MACE, NSTEMI on day 30. This patient was diagnosed with undifferentiated chest pain and discharged home from the ED with advice to follow-up with own cardiologist. There was a total of 7 patients (0.4%) who died in the study cohort within 30-days. Six patients died within 15-days: two patients died due an unknown cause, two died because of NSTEMI and two more died due to end stage heart failure. One additional patient died on day 16 due to heart failure. Nine patients (0.5%) suffered non-ACS outcomes (Table 6) within 15-days related to the presenting symptom, 8 patients were diagnosed with pulmonary embolism and 1 patient with underlying severe aortic stenosis requiring surgery. None suffered non-ACS outcomes after 15-days.

**Table 6:** Secondary outcomes among study patients–N = 1,683.

Outcome, n (%)	Total
MACE between days 16 and 30	1*
Deaths not related to cardiac ischemia	3^†^
Non-ACS outcomes^+^	9 (0.5)

*This patient had two TNI measurements 3-hours apart on the index visit and both were negative and then went on to suffer NSTEMI on day 30

^†^All three patients died due to end stage heart failure

^+^ Non-ACS (Acute Coronary Syndrome) outcomes include 8 patients with pulmonary embolism and 1 patient with severe aortic stenosis requiring surgery and all occurred before 15-days

## Discussion

In this prospective cohort study, we developed a pathway, the Ottawa Troponin Pathway for accurate identification of patients at-risk for 15-day MACE based on two cTnI measurements using Siemens Vista conventional assay 3-hours apart. The pathway involves a step-wise approach to troponin testing for patients with suspected NSTEMI in whom unstable angina has been ruled out by initial clinical evaluation and ECG. Using the absolute values or relative changes in values between the two cTnI measurements, the Ottawa Troponin Pathway would enable physicians to accurately rule-in or rule-out NSTEMI among majority of patients presenting to the ED with ACS symptoms. Patients identified as higher risk by the OTP will need referral to the admitting service such as cardiology in ED with a presumptive working diagnosis of NSTEMI.

In our study, overall 5.2% of patients suffered 15-day MACE with 5.1% suffering NSTEMI and 0.1% deaths due to cardiac ischemia or an unknown cause. Our study results are comparable to the AMI and death rates in a previous prospective study conducted at our study sites.[16] In our study among those who suffered 15-day MACE, there were 2 patients who died due to an unknown cause. Both patients had underlying coronary artery disease and further medical records review suggested that cardiac ischemia is more likely the cause of death in these patients.

There were two previous studies that evaluated the ability of two serial conventional cTn measurements 3-hours apart by secondary analysis to rule out short-term MACE.[10, 11] Mahler et al. conducted a secondary analysis of 1,005 patients enrolled at 18 US sites for the Myeloperoxidase In the Diagnosis of Acute coronary syndromes Study (MIDAS) study and found that negative conventional cTnI assay at 0 and 3 hours ruled out ACS among patients with a low HEART (includes History, ECG, Age, Risk factors and Troponin) score.[11] By a randomized trial Mahler et al. compared the HEART pathway to usual care.[10] The HEART pathway combines the use the HEART score and serial cTnI testing at 0 and 3 hours. In this study serial troponin testing using a similar Siemens assay, 30-day MACE was ruled out by negative serial conventional cTnI assay at 0 and 3 hours among patients with low HEART score. In our study, the addition of HEART score improved the sensitivity minimally at the cost of substantial reduction in specificity. When compared to a similar study published by Chapman et al., there was a similar reduction is specificity that was reported and a modest improvement in sensitivity.[17] It is possible that exclusion of patients with unstable angina could have led to different results in our study.

Our pathway is specifically developed for use with the Siemens Vista conventional cTnI assay and the utility will likely decrease as more institutions adopt hs TnI assays. However, comparative studies have shown no improved benefit of the hs TnI assay over the conventional assay. The authors in HEART pathway randomized study, compared the test characteristics of conventional and hs TnI assays and found no added benefit from the hs TnI assay.[10] Another randomized Australian trial found that hs TnI assay did not improve clinical outcomes for MACE at 1-year when compared to convention cTnI assay.[6] As the main objective of our study is to asses the absolute or relative changes in cTnI levels for diagnosis of NSTEMI, our study protocol did not mandate the formal use of risk scores such as HEART or TIMI (Thrombolysis in Myocardial Infarction) risk scores by the treating physicians. No previous studies have been conducted to show that use of these risk scores improve MACE detection when compared directly to clinical judgement. In Canada in general and at the study sites specifically, there are no chest pain observation units and further provocative stress tests are administered either through outpatient follow-up or hospitalization. The recent American College of Emergency Physicians’ clinical policy on management of ED patients with non-ST-elevation ACS discourages the routine use of further diagnostic testing such as stress test or computed tomography angiography prior to discharge in low-risk patients.[2]

Our study does have some limitations, 13.7% of patients had TnI measurements before the 3-hour mark. However, these patients did not suffer from MACE misdiagnosis. We were unable to include 20.5% patients in the study cohort for pathway development as they had only one troponin measurement. The decision to perform only one measurement among these patients is likely because of prolonged time lag from the time of recent onset of symptoms and the one patient with NSTEMI in this subgroup was appropriately diagnosed (Fig 1). The ED LOS was substantially lower in this subgroup when compared to patients who had two or more cTnI measurements performed. The prevalence of NSTEMI (5.2%) in our cohort is lower than previously reported and hence, caution must be exercised in settings with higher prevalence. However, there are huge variations in proportion of patients with acute myocardial infarction reported in previously published studies, ranging from 4.5% to 43.8%.[18] We were unable to complete 30-day follow-up for approximately 4% of patients. Given that a low proportion of patients suffered MACE and such a small number of patients were lost to follow-up, these patients are unlikely to influence the results of the study. The sensitivity of the pathway to identify patients for 15-day MACE was 99% with a 95% confidence interval around this estimate ranging from 94% to 100%. The width of this confidence interval is relatively wide due to the low event rate, i.e., only 88 patients with 15-day MACE. A sample size more than three times as large will be needed to achieve tighter intervals around this estimate. For example, a sample size of 300 patients with 15-day MACE (or a cohort size of 6000 based on an expected incidence of 5%) would be required to estimate an exact binomial confidence interval around the sensitivity of 99% with lower limit not dropping below 97%. Additionally, the number of patients in some subgroups, particularly the group with one of first two troponin values >45 but <100ng/L was very small resulting in wide confidence interval for the point estimate for sensitivity. While we calculated the HEART score with the best available information in the chart, we must acknowledge that some of the components for atherosclerosis risk such as cigarette smoking, family history and obesity were not reliably documented.

Our study was the first study to develop a pathway for ED management of patients with ACS symptoms based on their troponin values. Such pathways are lacking for many of the troponin assays currently in clinical use, particularly guidelines for management of patients with values above the 99^th^ percentile cut-off. The OTP was able to accurately classify patients in our study except for one patient who suffered NSTEMI. This was a 69 years old patient with extensive underlying coronary artery disease diagnosed with unstable angina on the index visit by the ED physician and referred to cardiology. The cardiology team disagreed and discharged the patient home, only for the patient to return after 4 days with ongoing symptoms at which time both the ED physician and the cardiologist agreed on the diagnosis of unstable angina and was hospitalized. This patient was diagnosed with NSTEMI while in-hospital and had catherization with one stent placement. We believe if unstable angina was properly diagnosed in this patient, our pathway would have achieved 100% sensitivity to identify 15-day MACE. Additionally, we have validated the pathway among patients with cTnI values >45 ng/L through health records review and have submitted for publication. The pathway should be validated before applying to other populations. We encourage investigators to develop similar pathway for other assays, particularly the hs cTn assays before widespread use. In our study, 71 patients (4.2%) had troponin elevations that classified these patients as high-risk as per the pathway, however, they did not suffer the outcome. We believe that the proportions of patients with such false positive elevations in troponin that are not associated with type-1 myocardial infarction will likely increase with the widespread use of hs cTn assays even with development of pathways like the OTP.

The OTP developed based on the results of this study cohort can accurately identify patients at-risk with very high sensitivity and specificity for 15-day MACE based on either absolute or relative changes between two cTnI measurements performed 3-hours apart. A small proportion of patients with intermediate cTnI values may need a third 6-hour cTnI measurement for rule-in or rule-out of NSTEMI. Application of the pathway has the potential to standardize troponin testing among patients with suspected ACS and reduce ED LOS at the study hospitals.

## Conclusions

In this prospective cohort study, using a large cohort of patients presenting to the ED with ACS symptoms, we developed the Ottawa Troponin Pathway. The pathway requires two conventional cTnI measurements performed 3-hours apart with a small proportion of patients with slight elevations above the 99^th^ percentile cut-off requiring a third measurement at 6-hours for accurate identification of patients at-risk for 15-day MACE. The addition of HEART score improved the sensitivity minimally with substantial reduction in specificity. The Ottawa Troponin pathway should lead to better identification of patients with NSTEMI, particularly those values above the 99^th^ percentile cut-off, and accurate yet quicker rule-in and rule-out of NSTEMI. Overall, this should improve ED crowding by reducing ED LOS, and efficient standardized ED management of patients with ACS symptoms.
